# Supplementary material for: Bedside Evaluation of Early VAS/NRS Based Protocols for Intravenous Morphine in the Emergency Department: Reasons for Poor Follow-Up and Targeted Practices
Source: J Clin Med. 2021 Oct 29;10(21):5089. doi: 10.3390/jcm10215089 (PMC8584399; doi:10.3390/jcm10215089)
Supplement: Supplementary file 1 [file jcm-10-05089-s001.zip › jcm-1410223-supplementary.pdf]

Journal of
Clinical Medicine

MDPI

Article

Supplementary Materials: Bedside Evaluation of Early VAS/NRS Based Protocols for Intravenous Morphine in the Emergency Department: Reasons for Poor Follow-Up and Targeted Practices

Virginie Eve Lvovschi, Karl Hermann, Frédéric Lapostolle, Luc-Marie Joly and Marie-Pierre Tavolacci

Table S1. Overlap between "real" and "simulated" conditions of prescription: description of the 41 concordant reasons in the subgroup analysis considering 79 non-titrated patients in both conditions.

	Etiologic Treatment	Renal Colic	Headache	NRS Subjective Reassessment by Physician	Prioritization of Another Class of Analgesics	Patient's Background	Minor Trauma	Total
Etiologic treatment	1	0	0	0	0	0	0	1
Renal colic	0	4	0	0	0	0	0	4
Headache	0	0	7	0	0	0	0	7
NRS subjective reassessment by physician	0	0	0	5	0	0	0	5
Prioritization of another class of analgesics	0	0	0	0	20	0	0	20
Patient's background	0	0	0	0	0	2	0	2
Minor trauma	0	0	0	0	0	0	2	2
Total	1	4	7	5	20	2	2	41

Lines present results in "real" conditions, columns present results in "simulated" conditions.

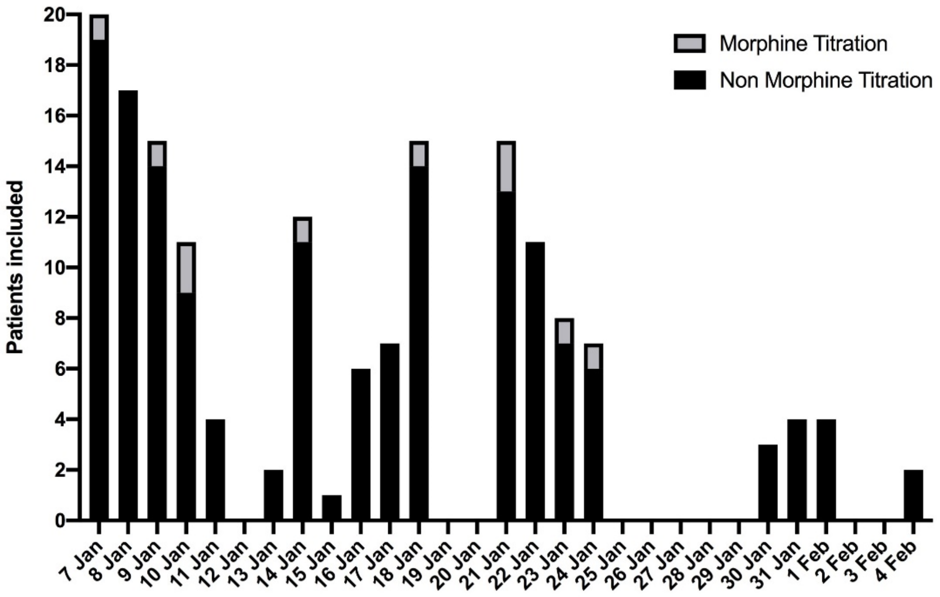

Figure S1. Inclusions in the cross-sectional study.
